# CDC25A governs proliferation and differentiation of FLT3-ITD acute myeloid leukemia

**DOI:** 10.18632/oncotarget.5706

**Published:** 2015-10-16

**Authors:** Sarah Bertoli, Helena Boutzen, Laure David, Clément Larrue, François Vergez, Anne Fernandez-Vidal, Lingli Yuan, Marie-Anne Hospital, Jérôme Tamburini, Cécile Demur, Eric Delabesse, Estelle Saland, Jean-Emmanuel Sarry, Marie-Odile Galcera, Véronique Mansat-De Mas, Christine Didier, Christine Dozier, Christian Récher, Stéphane Manenti

**Affiliations:** ^1^ Cancer Research Center of Toulouse, Inserm UMR 1037, CNRS ERL 5294, Université de Toulouse, Oncopole, Toulouse, France; ^2^ Hematology Department, Institut Universitaire du Cancer Toulouse–Oncopole, Toulouse, France; ^3^ Hematology Laboratory, Institut Universitaire du Cancer Toulouse–Oncopole, Toulouse, France; ^4^ Institut Cochin, Université Paris Descartes, CNRS UMR 8104, INSERM U 1016, Paris, France; ^5^ IPSEN Laboratory, Les Ulis, France

**Keywords:** cell cycle, acute myeloid leukemia, differentiation, proliferation, CDC25A

## Abstract

We investigated cell cycle regulation in acute myeloid leukemia cells expressing the FLT3-ITD mutated tyrosine kinase receptor, an underexplored field in this disease. Upon FLT3 inhibition, CDC25A mRNA and protein were rapidly down-regulated, while levels of other cell cycle proteins remained unchanged. This regulation was dependent on STAT5, arguing for FLT3-ITD-dependent transcriptional regulation of CDC25A. CDC25 inhibitors triggered proliferation arrest and cell death of FLT3-ITD as well as FLT3-ITD/TKD AC-220 resistant cells, but not of FLT3-wt cells. Consistently, RNA interference-mediated knock-down of CDC25A reduced the proliferation of FLT3-ITD cell lines. Finally, the clonogenic capacity of primary FLT3-ITD AML cells was reduced by the CDC25 inhibitor IRC-083864, while FLT3-wt AML and normal CD34+ myeloid cells were unaffected. In good agreement, in a cohort of 100 samples from AML patients with intermediate-risk cytogenetics, high levels of CDC25A mRNA were predictive of higher clonogenic potential in FLT3-ITD+ samples, not in FLT3-wt ones.

Importantly, pharmacological inhibition as well as RNA interference-mediated knock-down of CDC25A also induced monocytic differentiation of FLT3-ITD positive cells, as judged by cell surface markers expression, morphological modifications, and C/EBPα phosphorylation. CDC25 inhibition also re-induced monocytic differentiation in primary AML blasts carrying the FLT3-ITD mutation, but not in blasts expressing wild type FLT3. Altogether, these data identify CDC25A as an early cell cycle transducer of FLT3-ITD oncogenic signaling, and as a promising target to inhibit proliferation and re-induce differentiation of FLT3-ITD AML cells.

## INTRODUCTION

Acute myeloid leukemia (AML) is characterized by increased proliferation and cell death resistance, and by a block of the hematopoietic process occurring at different stages of the myeloid differentiation [1]. The impact of several mutations has been explored this last decade, one of the most frequent being the internal tandem duplication (ITD) in the juxta-membrane domain of the Fms-Like Tyrosine kinase 3 (FLT3), which leads to constitutive activation of this receptor [2]. This mutation is particularly associated to normal karyotype [3] and now takes part to the most recent prognostic classification of AML [4]. During normal myeloid hematopoiesis, FLT3 is highly expressed and was reported to play an important role at the granulo-monocyte progenitor level [5].

Because of the high frequency of this mutation (25–30% of AML) and of its associated negative prognostic [3, 6], several FLT3 inhibitors have been consequently developed and tested in different clinical trials, either as single agent or in combination with chemotherapy [7–12]. These molecules have a negative impact on AML cells proliferation *in vivo*, and interestingly, their pro-differentiation effect was also reported clinically, suggesting that inhibiting FLT3-ITD could partially relieve the differentiation arrest occurring in this category of AML [13]. Recent studies identified the ERK kinase and the cyclin-dependent kinase CDK1 as important players of FLT3-ITD AML differentiation arrest through phosphorylation of the C/EBPα transcription factor on its serine 21 [14–16], suggesting that CDK or ERK inhibitors could restore the differentiation program of these cells.

CDC25A is a dual specificity phosphatase involved in cyclin-dependent kinases activation during the cell cycle. CDC25A has important functions during replication and mitosis, as well as during the G1 phase of the cell cycle. CDC25A is finely regulated both at the transcription and protein levels [17], and moderate variations of its cellular level affect genomic stability and oncogenic transformation process [18]. CDC25A knock-out is lethal at an early stage of embryonic development. Its overexpression was described in different categories of cancers, and was often associated with an adverse prognosis [19]. However, there is almost no study dealing with CDC25A status in AML or in other myeloid malignancies. CDC25A expression is increased by leukemic cells adhesion to fibronectin, and participates to the adhesion-dependent increased proliferation of these cells [20]. CDC25A is also constitutively expressed downstream of oncogenic tyrosine kinases, including NPM-ALK and BCR-ABL [21], as well as JAK2 V617F in myeloproliferative neoplasms [22].

In this work, we demonstrate that CDC25A is an early target of FLT3-ITD oncogenic signaling, and is an important player of AML cells proliferation and differentiation arrest.

## RESULTS

### CDC25A is an early target downstream of FLT3-ITD

**Figure 1:**
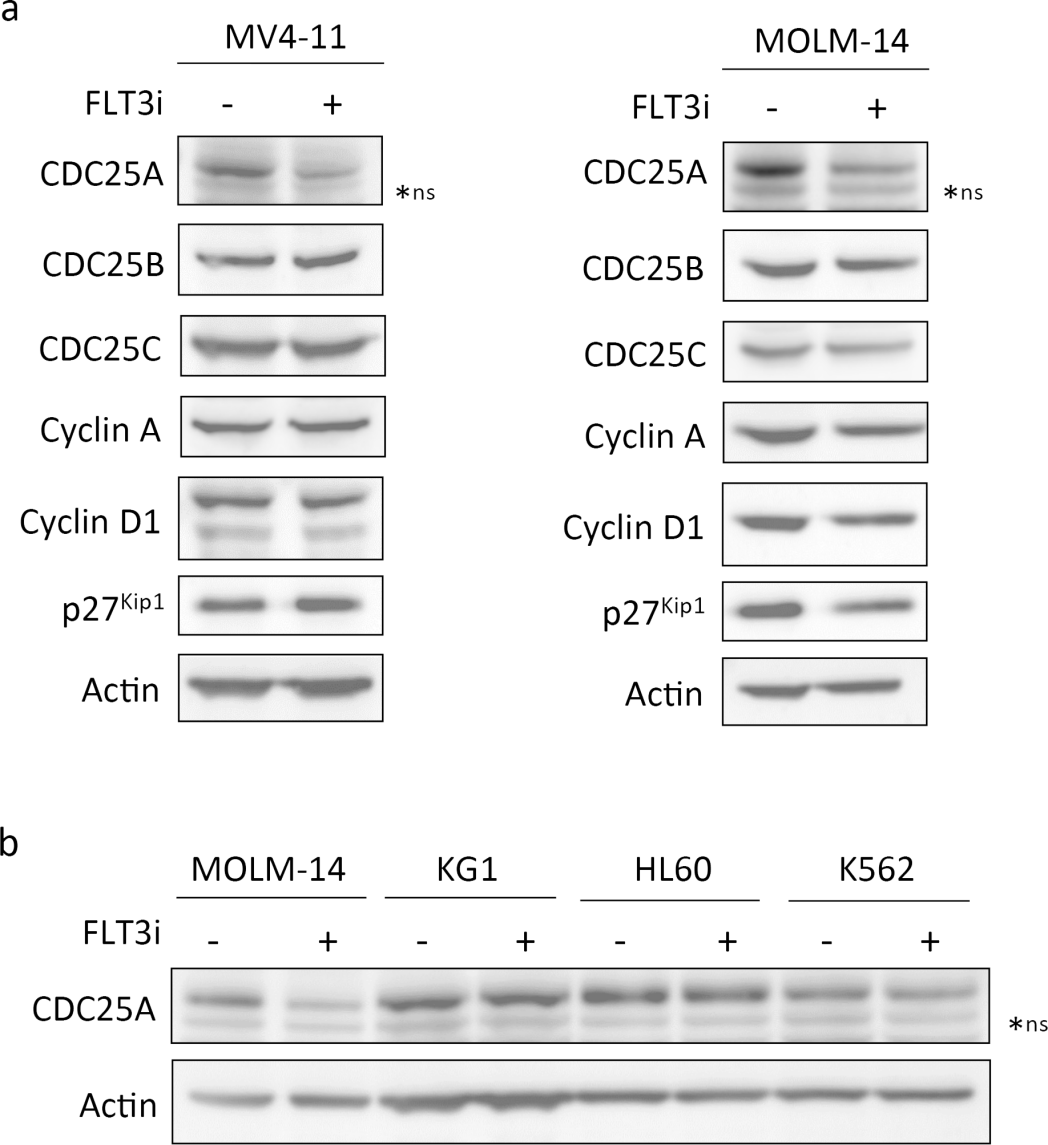
CDC25A is an early cell cycle target downstream of FLT3-ITD **a.** MV4-11 (left panel) and MOLM-14 (right panel) FLT3-ITD expressing cells were treated for 2 hours with FLT3 inhibitor III (100 nM), and the protein levels of CDC25A, CDC25B, CDC25C, Cyclin A, Cyclin D1 and p27^Kip1^ were analyzed by western blot. **b.** MOLM-14 (FLT3-ITD positive), KG1 and HL-60 (FLT3wt positive) and K562 (FLT3 negative) cell lines were treated for 2 hours with FLT3 inhibitor III and the level of CDC25A was analyzed by western blot. Actin was used as a loading control. These results are representative of three independent experiments. ns: non specific.

In order to identify links between the FLT3-ITD mutated receptor and cell cycle progression, we investigated the expression of cell cycle regulating proteins upon FLT3-ITD inhibition in MV4-11 and MOLM-14, two AML cell lines carrying FLT3-ITD mutation. Two unrelated pharmacological inhibitors of FLT3 (FLT3 inhibitor III and the potent new generation inhibitor AC220, quizartinib) induced CDC25A down-regulation in these cell lines (Figure 1a and Supplementary Figure S1a) in a dose-dependent manner (Supplementary Figure S1b). Neither other members of the CDC25 phosphatase family CDC25B and CDC25C, nor key cell cycle regulators as cyclin A, cyclin D1 or p27^Kip1^ were significantly modified in these conditions (Figure 1a). Cell cycle distribution of AML cells was not changed after two hours in the presence of FLT3 inhibitor III (not shown), indicating that CDC25A down-regulation was not a consequence of cell cycle arrest. The efficiency of FLT3 inhibitors in FLT3-ITD expressing cell lines was ascertained by the inhibition of Akt, STAT5 and ERK phosphorylation, three pathways activated downstream of FLT3-ITD (Supplementary Figures S1a and S1c). In good agreement, we also observed CDC25A down-regulation in response to FLT3-ITD inhibition in the murine cell line BaF3 constitutively expressing FLT3-ITD (Supplementary Figure S1d). Importantly, FLT3 inhibitor did not decrease CDC25A protein level in FLT3-wild type leukemic cell lines KG1 and HL-60 or in the FLT3 non-expressing cell line K562 (Figure 1b).

### Mechanisms of CDC25A regulation downstream of FLT3-ITD

**Figure 2:**
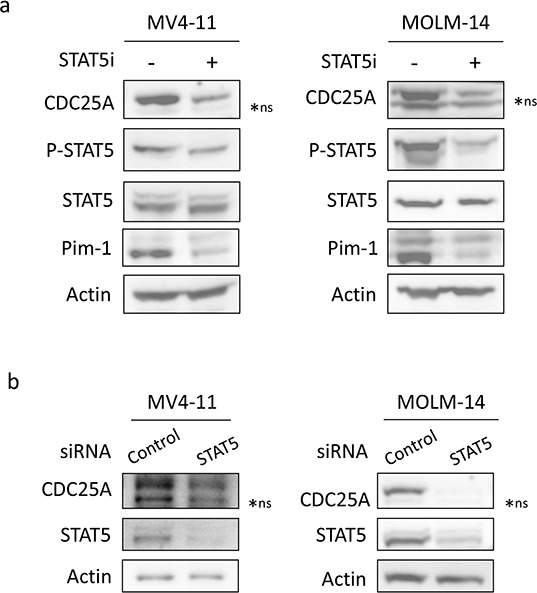
STAT5 regulates CDC25A downstream of FLT3-ITD **a.** MV4-11 (left panel) and MOLM-14 (right panel) cells were treated for 2 hours with STAT5 inhibitor (100 nM). CDC25A, Pim1, and STAT5 protein and phosphorylation levels were analyzed by western blot. **b.** MV4-11 and MOLM-14 cells were transfected for 24 hours with STAT5A/B siRNA and the impact on CDC25A protein level was analyzed by western blot. These results are representative of three independent experiments. Actin was used as a loading control. ns: non specific.

We then investigated which signaling pathway was involved in CDC25A regulation. As shown in Figure 2a, a STAT5 inhibitor significantly reduced CDC25A protein level in MV4-11 and MOLM-14 cells after 2 hours. This was not the case in KG1 and HL-60 cells (Supplementary Figure S2a), nor in TF-1 cells, another leukemic cell line expressing wild type FLT3, upon different culture conditions, including stimulation by GM-CSF, IL-3 or G-CSF (Supplementary Figure S2b). By contrast to STAT5 inhibition, neither Akt nor ERK inhibition induced such down-regulation of CDC25A in FLT3-ITD positive cell lines (Supplementary Figure S2c and S2d), suggesting that STAT5 is the major pathway leading to CDC25A regulation downstream of FLT3-ITD. In good agreement with these data, siRNA-mediated STAT5 knock-down induced CDC25A protein down-regulation in MOLM-14 and MV4-11 cells, confirming the results observed with pharmacological inhibition (Figure 2b).

**Figure 3:**
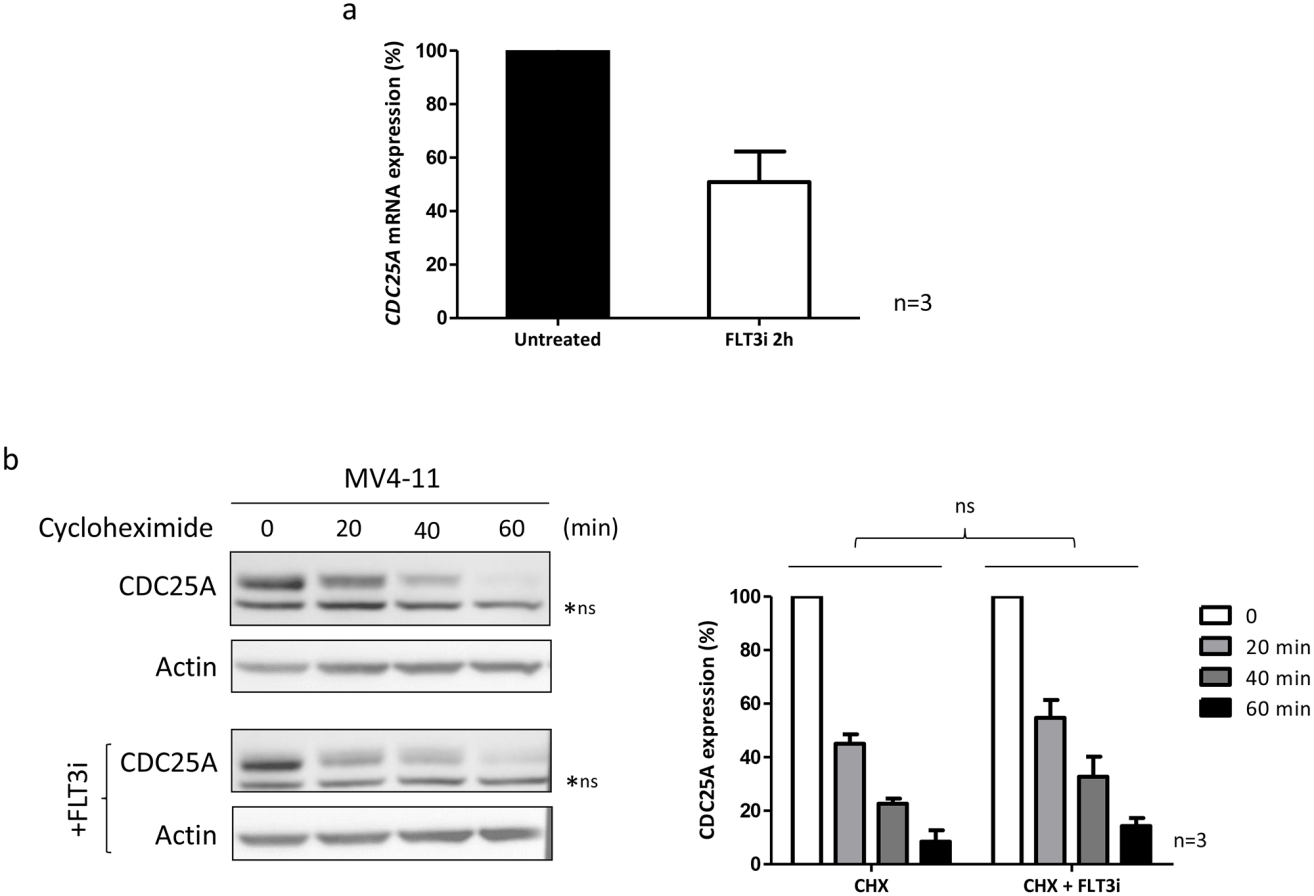
FLT3-ITD regulates CDC25A mRNA level **a.** *CDC25A* mRNA expression was measured in MOLM-14 cells by quantitative RT-PCR after FLT3 inhibition for 2 hours (100 nM). The graph shows the mean +/− SEM of *CDC25A* mRNA expression in untreated and treated cells, in three independent experiments. **b.** MV4-11 cells were treated with cycloheximide (50 μg/mL) for the indicated times. FLT3 inhibitor III (100 nM) was added 30 minutes before cycloheximide treatment, and left in the medium. This western blot is representative of three independent experiments. The right panel shows the quantification of three independent experiments. ns: non specific.

Since STAT5 is a transcription factor, we then estimated variations of *CDC25A* mRNA level by quantitative RT-PCR in response to FLT3-ITD inhibition. Inhibiting FLT3-ITD for two hours significantly reduced *CDC25A* mRNA level in MOLM-14 cells (Figure 3a), suggesting that STAT5 could be a transcriptional regulator of CDC25A downstream of FLT3-ITD. Since modifications of CDC25A protein stability have been often described, we measured half-life of CDC25A protein in the presence of cycloheximide (Figure 3b). The rates of CDC25A down-regulation were similar in the presence or the absence of FLT3 inhibitor, indicating that FLT3-ITD does not significantly increase the stability of CDC25A in this model. Altogether, these data suggest that CDC25A transcription is regulated by STAT5 down-stream of FLT3-ITD.

### CDC25A is an important determinant of FLT3-ITD cells proliferation

**Figure 4:**
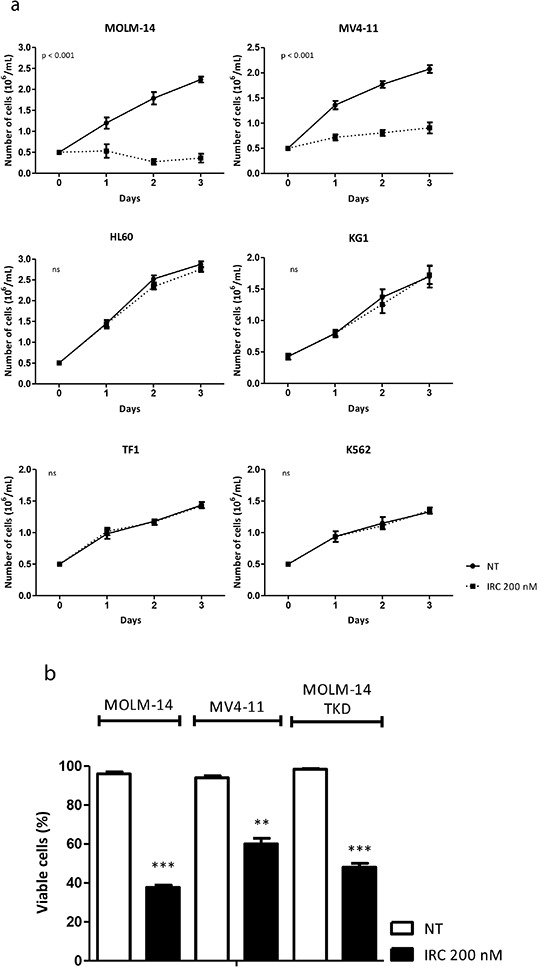

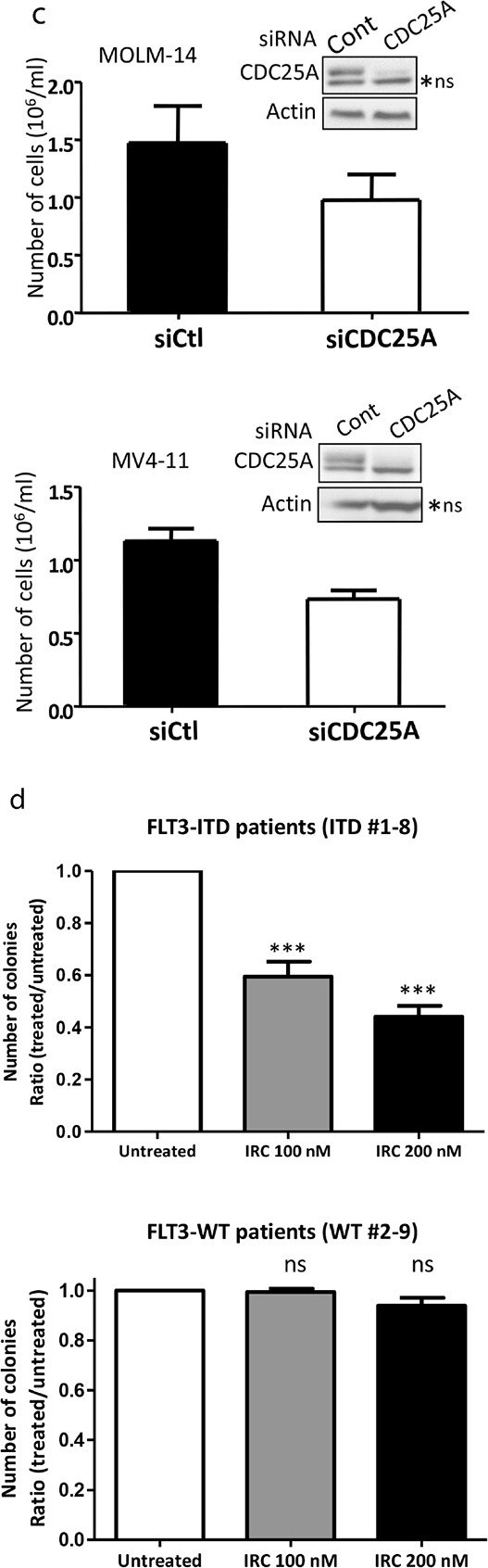

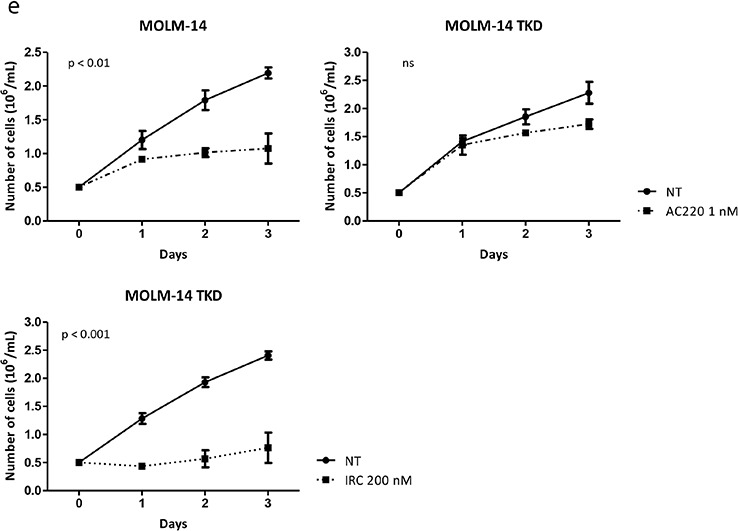
CDC25A is an important determinant of FLT3-ITD leukemic cells proliferation **a.** MV4-11 and MOLM-14 FLT3-ITD positive cells, KG1, HL-60 and TF-1 FLT3 wild type cells, and K562 FLT3 negative cells were cultured in the presence of the CDC25 inhibitor IRC-083864 (200 nM). Cells were harvested each day and counted after trypan blue staining. The graph represents three independent experiments. **b.** Cell death was estimated by trypan blue staining in cells (MOLM-14; MV4-11; MOLM-14 TKD) treated with IRC-083864 200 nM for 48 hours. **c.** MOLM-14 and MV4-11 cells were transfected with CDC25A siRNA for 24 hours, and cells were counted after trypan blue coloration. The efficiency of CDC25A siRNA was estimated by western blot analysis (inserts). ns: non specific. **d.** Primary cells from patients were cultured in semi-solid medium to estimate their clonogenic potential as described in the Methods section, in the presence or the absence of IRC-083864 (100 and 200 nM). 8 FLT3-ITD positive (upper panel) and 8 FLT3-wild type (lower panel) AML primary samples were used for these experiments. Leukemic colonies were scored under an inverted microscope at day 7. **e.** MOLM-14, and MOLM-14 TKD cells, generated as described in Supplementary material and methods, were grown in the presence of AC-220 1 nM (upper panel) or IRC-083864 200 nM (lower panel). Cells were harvested each day and counted after trypan blue staining. The graphs represent three independent experiments.

To ask whether FLT3-ITD cells are dependent of CDC25A activity for their proliferation, we first used IRC-083864, a potent pharmacological inhibitor of CDC25 (A, B and C) previously characterized *in vitro* and *in vivo* in different cancer models [23]. IRC-083864 inhibited the proliferation of MV4-11 and MOLM-14 cell lines, but did not decrease the proliferation of KG1, HL-60, TF-1 and K562 control cells (Figure 4a). In addition, IRC-083864 had no effect on KG1 and HL60 cells proliferation upon stimulation by FLT3 ligand (Supplementary Figure S3a). As shown in Figure 4b, IRC-083864 induced significant cell death in MV4-11 and MOLM-14, but did not induce accumulation in any specific phase of the cell cycle (not shown). Since this inhibitor has similar efficiencies on CDC25A, CDC25B and CDC25C, we then performed RNA interference experiments to estimate the specific impact of CDC25A on FLT3-ITD+ cells proliferation. As shown in Figure 4c and in Supplementary Figure S3b, RNA interference-mediated down-regulation of CDC25A, performed with two independent siRNA targeting the coding region or the 3′-UTR, reduced proliferation of MOLM-14 and MV4-11 cells. Importantly, similar down-regulation of CDC25A had negligible effect on KG1 cells (Supplementary Figure S3c), confirming that FLT3-ITD positive cells are more dependent on CDC25A than FLT3-ITD negative ones.

We then measured the impact of CDC25 inhibition on the proliferation of AML primary cells from patients. The main biological characteristics of the samples used in these experiments are depicted in Table 1. The clonogenic potential of FLT3-ITD primary cells in semi-solid 5637-conditionned medium was significantly reduced in the presence of IRC-083864 in a dose-dependent manner (Figure 4d, upper panel), while cells expressing wild-type FLT3 (Figure 4d, lower panel) were not sensitive to CDC25 inhibition. Similar results were obtained with a semi-solid medium containing recombinant growth factors GM-CSF, FLT3 ligand, and IL-3 (Supplementary Figure S3d). In addition, the IC-50 of IRC-083864 on CD34+ cells purified from bone marrow of healthy donors was >500 nM (not shown), much more higher than for FLT3-ITD AML cells (see Figure 4d). Taken together, these data demonstrate that leukemic cells expressing FLT3-ITD are highly dependent on CDC25A for their proliferation.

**Table 1:** Biological properties of primary AML samples

Sample	Gender	Age (years)	FAB	AML status	WBC (×10^9^/L)	Karyotype	*FLT3*	*NPM1*	Outcome
ITD #1	F	82	5	DN	83	Normal	ITD 30%	*NPM1c*	Decitabine–Treatment failure
ITD #2	M	67	NA	NA	NA	Normal	ITD 27%	*NPM1c*	NA
ITD #3	M	63	4	S	183	Normal	ITD 45%	WT	Intensive CT–relapse 2 mo after CR1 - AlloSCT in CR2
ITD #4	F	79	2	*DN*	36	t(6;9)	ITD 18%	WT	Best supportive care
ITD #5	M	12	2	*DN*	NA	t(6;9)	ITD 69%	WT	Intensive CT–Alive in CR
ITD #6	M	38	1	*DN*	47	Normal	ITD 24%	WT	Intensive CT–Alive in CR
ITD #7	F	65	4	*DN*	161	Normal	ITD	WT	Intensive CT-Relapse 18 mo after CR
ITD #8	M	68	1	*DN*	38	Normal	ITD	*NPM1c*	Intensive CT–Alive in CR after 6 yrs
WT #1	F	58	1	*DN*	60	del(7p)	WT	WT	Intensive CT–Alive in CR
WT #2	F	82	1	NA	20	Normal	WT	*NPM1c*	Best supportive care
WT #3	F	66	2	S	54	Normal	WT	*NPM1c*	Intensive CT-Relapse 5 mo after CR
WT #4	M	79	2	S	19.5	Normal	WT	WT	Best supportive care
WT #5	F	51	1	*DN*	88	Normal	WT	*NPM1c*	Intensive CT–Alive in CR
WT #6	F	81	NA	*DN*	422	Normal	WT	WT	Intensive CT–Died in aplasia
WT #7	M	54	2	*DN*	24	Normal	WT	*NPM1c*	Intensive CT–Alive in CR
WT #8	M	83	1	S	147	Adverse 3 and 7	WT	WT	Intensive CT–Treatment failure
WT #9	M	76	4	*DN*	20	Normal	WT	WT	Best supportive care

A summary of the main biological properties of primary AML samples used in Figure 4, Figure 6, and Supplementary Figure S5. Gender, age, French-American-British (FAB) classification, AML status (ie. de novo or secondary), white blood cell count at diagnosis, karyotype, FLT3-ITD (with allelic ratio when mutated) and NPM1 mutational status are listed, as well as outcome features. AlloSCT: allogeneic stem cell transplantation; CR: complete remission; CT: chemotherapy; DN: de novo; F: female; M:male; mo: months; NA: non available; WT: wild-type; NPM1c: NPM1 cytoplasmic, ie. mutated; S: secondary; WBC: white blood cell count.

### FLT3-ITD/TKD expressing cells are resistant to AC220 but remain sensitive to CDC25 inhibition

In recent clinical trials performed with pharmacological FLT3 inhibitors such as quizartinib or sorafenib [24], heterogeneous mechanisms were suggested to contribute to FLT3 inhibitors resistance, and among them FLT3 kinase domain mutations were the most frequently reported [25]. We consequently developed a cellular model consisting of MOLM-14 cells transfected with a FLT3-ITD mutant with a D835Y amino-acid substitution within the FLT3 kinase domain (FLT3-ITD-D835Y; FLT3-ITD/TKD) (see Supplementary Methods). In MOLM-14 and in MOLM-14 expressing FLT3-ITD/TKD, treatment with 1nM AC220 induced low levels of cell death (not shown), and as expected, ITD/TKD cells were resistant to AC220 by comparison with parental MOLM-14 (Figure 4e; upper panel). By contrast, 200 nM IRC-083864 induced cell proliferation arrest and cell death similar to that observed with MOLM-14 and MV4-11 cell lines (Figure 4b; lower panel), suggesting that in some circumstances CDC25 inhibition could overcome resistance to FLT3 inhibitors.

### CDC25A level predicts clonogenic capacity of FLT3-ITD primary cells

We then investigated the expression level of CDC25A mRNA in a cohort of 188 non-promyelocytic AML young patients (aged 18–65) treated by intensive chemotherapy in Toulouse University Hospital in the 2000–2010 period. The characteristics of this cohort are detailed in Supplementary Methods and in Supplementary Tables S1–S3. CDC25A mRNA expression was divided into low expression and high expression according to the median value of the entire cohort. We observed no difference in the expression of CDC25A between FLT3-wt and FLT3-ITD patients. FLT3-ITD allelic ratio or insertion length were also not determinants for CDC25A expression. Since CDC25A appears as an important actor of cell proliferation in FLT3-ITD cells (see Figure 4), we looked for correlations between CDC25A expression and clonogenic potential in FLT3-ITD patients. Clonogenic assays were performed at diagnosis for 151 patients. We first restricted our analysis to the intermediate-risk cytogenetic group (*n* = 100) where FLT3-ITD has its prognostic significance. As shown in Table 2, high CDC25A mRNA levels nicely correlate with higher clonogenic potential in FLT3-ITD patients, while this is not the case in FLT3-wt ones. Similar results were obtained in the normal karyotype subgroup (*n* = 67). These data suggest that high CDC25A expression confers proliferation advantage to FLT3-ITD positive cells.

**Table 2:** Clonogenic properties of AML cells according to *CDC25A* mRNA expression

	Low CDC25Amedian (IQR)	High CDC25Amedian (IQR)	p*
Total intermediate karyotype (*n* = 100)	850 (0–7850)	2400 (150–10800)	0.16
Intermediate karyotype with *FLT3*-ITD (*n* = 35)	200 (0–1240)	5575 (2200–17850)	0.03
Intermediate karyotype with *FLT3*-WT (*n* = 59)	2225 (0–11650)	1100 (100–10800)	0.76
Normal karyotype with *FLT3*-ITD (*n* = 31)	650 (0–4650)	5250 (1975–12638)	0.09
Normal karyotype with *FLT3*-WT (*n* = 36)	2550 (0–8050)	3800 (250–11850)	0.45

* Comparisons between low CDC25A and high CDC25A subgroups were performed using Mann-Whitney *U* test

Values are expressed in number of colonies per 10^6^ cells.

### CDC25A is involved in FLT3-ITD AML cells differentiation arrest

**Figure 5:**
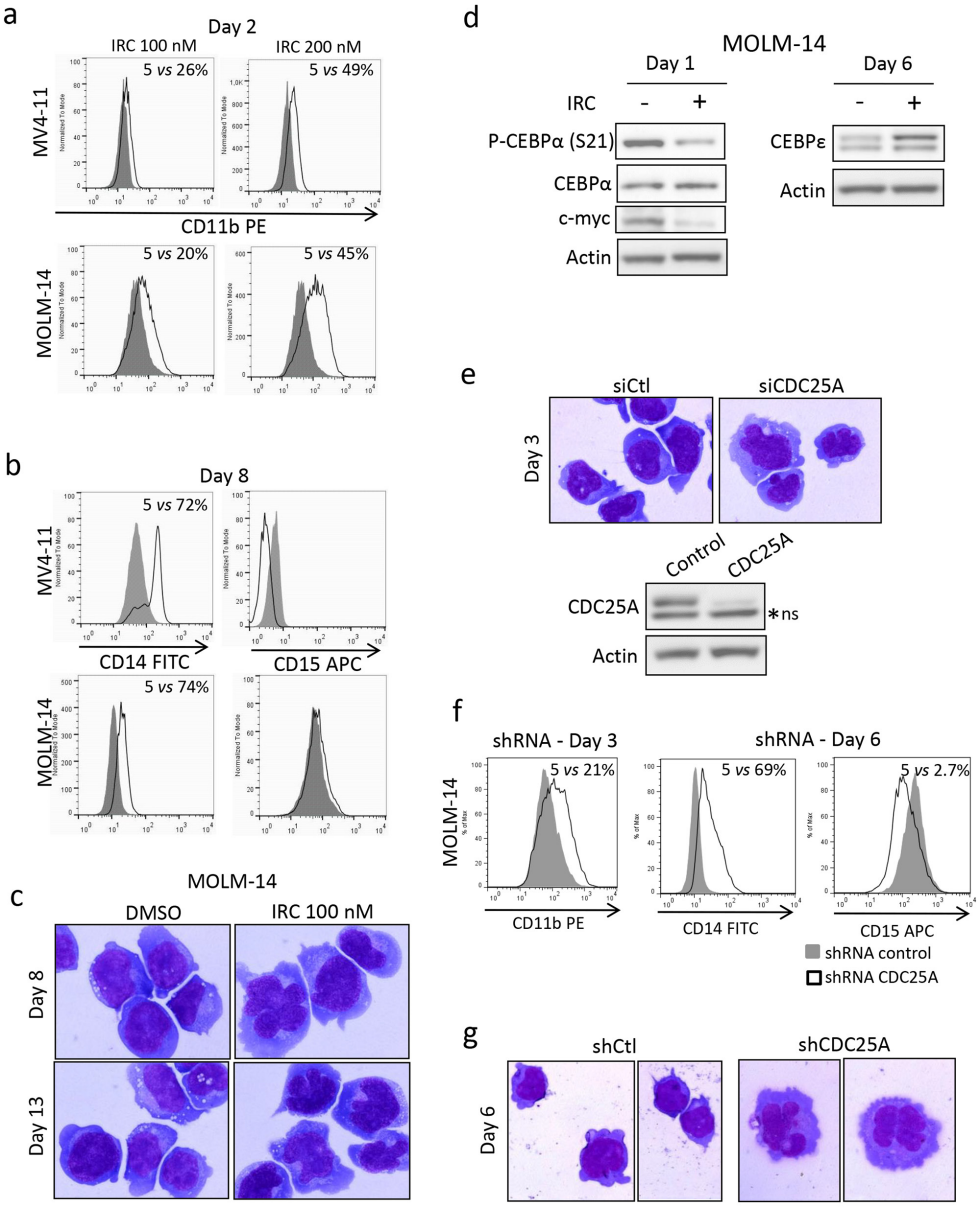
CDC25 inhibition relieves differentiation block in FLT3-ITD AML cell lines **a–b.** MOLM-14 and MV4-11 cells were treated for different times with IRC-083864 and the expression of the cell surface markers CD11b, CD14 and CD15 was analyzed by flow cytometry or **c.** by microscopy after May-Grünwald-Giemsa staining. Original magnification x100. **d.** MOLM-14 cells were treated with IRC-083864 (100 nM). c-myc expression as well as C/EBPα ser 21 phosphorylation were analyzed by western blot at day 1, and C/EBPε expression at day 6. **e.** MOLM-14 cells were transfected with CDC25A siRNA, and their morphology was analyzed by microscopy after 3 days (upper panel). Original magnification x100. The efficiency of CDC25A siRNA was assessed by western blot analysis (lower panel). These results are representative of three independent experiments. ns: non specific. **f–g.** MOLM-14 cells were transfected with CDC25A shRNA. Expression of CD11b, CD14 and CD15 was followed by flow cytometry analysis at days 3 and 6 (f), and their morphology was analyzed by microscopy after 6 days (g). Original magnification x100.

A role for the cyclin-dependent kinase CDK1 in FLT3-ITD positive cells differentiation arrest was recently reported [16]. Since CDK1 is a major substrate of CDC25 phosphatases during mitosis, we reasoned that CDC25A could be a master regulator of leukemic cells differentiation through its CDK1 activating function. To test this hypothesis, MV4-11 and MOLM-14 cells were treated with lower dose 100 nM IRC-083864, and the differentiation of these cells was followed by cell surface markers expression and by morphological modifications at different times. As shown in Figure 5a, expression of the early granulo-monocytic marker CD11b was induced in a dose dependent manner as early as two days after CDC25 inhibition. After 8 days of treatment, the monocytic marker CD14 was increased in both cell lines (Figure 5b) while the granulocytic marker CD15 either decreased or did not change significantly at that time. These data suggest that CDC25 inhibition relieves the differentiation block and drives a monocytic differentiation process in FLT3-ITD expressing cell lines. Morphological analyses revealed monocytic-like nuclear changes in cells treated with IRC-083864 for 8 and 13 days (Figure 5c), consistent with modifications of surface markers expression. In good agreement, IRC-083864 also induced dephosphorylation of C/EBPα on serine 21, a phosphorylation catalyzed by CDK1 and/or ERK and involved in the differentiation arrest of these cells (Figure 5d). CDC25 inhibition also induced c-myc down-regulation at day 1 and C/EBPε up-regulation at day 6, two additional markers of myeloid differentiation. As a confirmation of these data, modifications of CD11b, CD14 and CD15 expression as well as nuclear morphological changes were also observed with another CDC25 inhibitor, NSC-95397 (Supplementary Figure S4).

In order to further confirm the role of CDC25A in this differentiation arrest, we performed RNA interference mediated down-regulation of the protein. We first used siRNA-mediated CDC25A down-regulation, which led to nuclear morphological modifications at day 3 in MOLM-14 cells (Figure 5e). To confirm these data, we performed lentiviral infection of MOLM-14 cells with an shRNA targeting CDC25A. As shown in Figure 5f, down-regulation of CDC25A induced CD11b and CD14 expression 3 and 6 days after infection respectively, while CD15 expression was decreased, confirming the data obtained by pharmacological inhibition and further arguing for the implication of CDC25A in the differentiation process. Consistently, morphological nuclear modifications were observed at day 6 after infection (Figure 5g).

**Figure 6:**
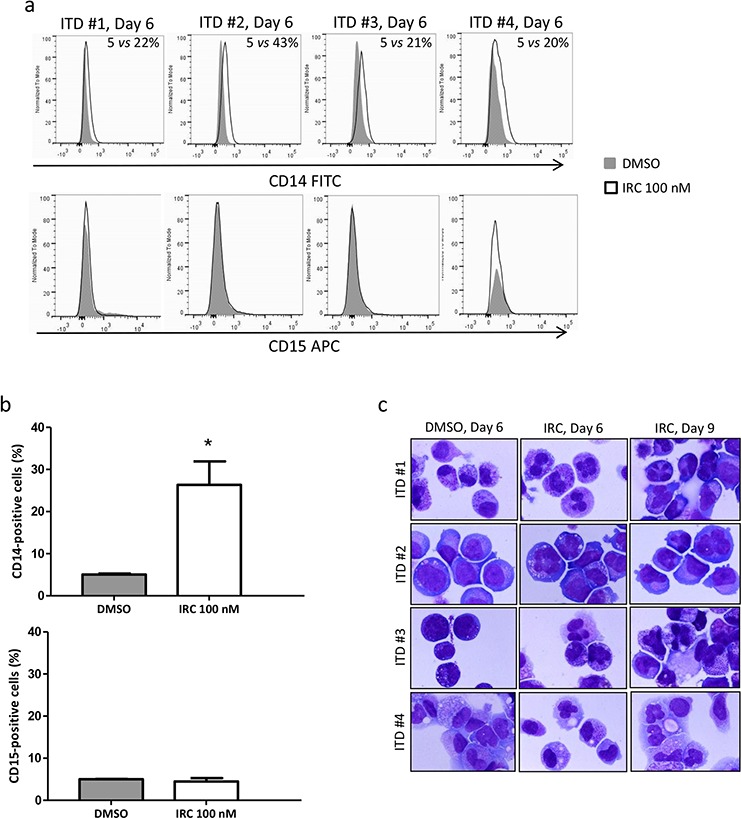
CDC25 inhibition relieves differentiation block in FLT3-ITD AML primary cells from patients **a.** 4 primary samples from patients carrying the FLT3-ITD mutation (ITD #1–4) were treated for 6 days with IRC-083864 (100 nM), and the expression of CD14 (upper panel) and CD15 (lower panel) was followed by flow cytometry analysis. **b.** Quantification (mean ± SEM, *n* = 4) of CD14 (upper panel) and CD15 (lower panel) expression in response to IRC-083864. **c.** The morphology of primary cells from patients used in (a) was analyzed by microscopy after 6 or 9 days of treatment with IRC-083864. Original magnification x100.

We then asked whether CDC25 inhibition could induce differentiation of primary AML samples expressing either FLT3-ITD or wild-type FLT3. As shown in Figure 6a and 6b, the monocytic-specific differentiation marker CD14 increased while CD15 expression was not modified after 6 days of treatment of FLT3-ITD primary cells with IRC-083864. Consistently, monocytic-like morphological nuclear changes were observed at days 6 and 9 of CDC25 inhibition in these FLT3-ITD expressing cells (Figure 6c). Similar results were obtained after treatment with the other CDC25 inhibitor NSC-95397 (Supplementary Figure S5a and S5b), while no evidence of differentiation was observed in FLT3-wild type samples in the same conditions (Supplementary Figure S5c and S5d).

Altogether these data demonstrate that inhibiting CDC25A reduces proliferation and induces monocytic differentiation of FLT3-ITD-positive AML cell lines and primary cells, and they argue for a central role of this phosphatase in the hematopoietic differentiation arrest of these cells.

## DISCUSSION

Because of high frequency and poor prognosis of FLT3-ITD mutation, improving the knowledge of this AML subgroup pathophysiology appears as an essential task for the next years. Major signaling pathways activated by FLT3-ITD have been identified, but downstream effectors, as well as their respective involvements in cell proliferation and drug resistance remain to be specified. Large phosphoproteomic analysis performed in FLT3-ITD expressing cells identified a panel of potential downstream targets of Pim and Akt, two well recognized players of FLT3-ITD oncogenic potential [26], but functional importance of these proteins in FLT3-ITD AML remained elusive. In this work, we demonstrate that the dual specificity phosphatase CDC25A, a major activator of cyclin-dependent kinases during different phases of the cell cycle, is regulated very early downstream of FLT3-ITD. Our data argue for a STAT5-dependent transcriptional mechanism being at the origin of this regulation, but we cannot rule out that CDC25A protein level is also governed at the translational level, as we recently observed down-stream of JAK2 V617F, another oncogenic tyrosine kinase involved in myeloproliferative disease [22]. Transcriptional regulation of CDC25A by STAT5 has not been reported up to now, but the involvement of STAT3 in both negative and positive transcriptional regulation of CDC25A has been described [27]. Different studies recognized the importance of STAT5 in FLT3-ITD signaling and leukemic cells transformation [28–30], and targeting of this pathway constitutes an important axis of therapeutic research [31]. Further experiments are needed to better understand CDC25A regulation by STAT transcription factors in AML cells.

One important feature of this work is that FLT3-ITD cells are highly dependent of CDC25A for their proliferation. This is true by comparison with FLT3-ITD negative leukemic cells, but also with normal CD34+ hematopoietic cells, opening the interesting hypothesis of a therapeutic window for CDC25 inhibitors. To this respect, the fact that CDC25 inhibition overcomes the resistance to FLT3 inhibitors induced by TKD mutation of FLT3-ITD appears of specific interest, and may constitute an interesting alternative to the development of new generation FLT3 inhibitors with higher efficiency on mutant forms of the receptor.

In this work, we also highlighted a central role for CDC25A in the differentiation process of FLT3-ITD AML subtype. Abnormalities of transcription factors-induced differentiation are observed in one third of AML (PML-RARA, AML1-ETO, CBFβ and C/EBPα). In particular, mutations in the C/EBPα gene are detected in 10% of AML with normal karyotype, and C/EBPα plays a key role in normal granulocytic or monocytic differentiation, depending on its dimerization partner [32]. Differentiation arrest in FLT3-ITD AML was recently described to be dependent on the phosphorylation of C/EBPα on serine 21 by the ERK kinase and/or the mitotic cyclin dependent kinase CDK1/cyclin B1 [16]. These authors proposed FLT3-ITD-dependent regulation of cyclin B1 protein as a key parameter of CDK1 activity, and consequent C/EBPα phosphorylation and differentiation arrest in this model. Our work suggests that CDC25A, in addition to cyclin B1, is another key regulator of CDK1 activity downstream of FLT3-ITD. This would suggest that FLT3-ITD up-regulates CDK1 activity by different ways, both by CDC25A-dependent dephosphorylation of Thr14/Tyr15, and by accumulation of cyclin B1 and its subsequent association with CDK1. The molecular mechanism of cyclin B1 accumulation in this context, and whether CDC25A activity could be involved in this process, remain to be established. CDC25A is involved in different phases of the cell cycle, and to this respect, is considered as a regulator of different CDKs. Its role as an activator of CDK2 and the G1/S transition and during DNA replication is well established, and its action on the G1 CDK4/CDK6-cyclin D1 complex has been recently highlighted [33]. Our data do not allow to distinguish which CDKs are regulated by CDC25A and involved in the differentiation process downstream of FLT3-ITD. In consequence, we cannot exclude that CDK2, and/or CDK4/CDK6 are important actors of this process. This hypothesis would be in line with the very recent identification of CDK6 as a critical effector of MLL fusions in leukemogenesis, underscoring that cell cycle regulators may have distinct, non-canonical, and non-redundant functions in different contexts [34]. By acting on different CDK/cyclin complexes, CDC25 inhibitors would constitute interesting candidates to increase the therapeutic tools in the AML personalized treatment, and intense research is ongoing to give rise to new potent compounds [35, 36]. Since a few works reported the possible importance of CDC25C [37] and CDC25B [38] in AML, CDC25 family members inhibition in these pathologies may be of interest in the future [39].

From a more general point of view, the ability of cells to escape terminal differentiation is one of the characteristics of cancer [40], and AML represents a paradigm for this phenomenon [41]. Reinducing leukemic cells differentiation constitutes an important alternative to genotoxic therapeutic agents currently used to treat these pathologies, but up to now, this approach is only routinely used in the case of promyelocytic leukemia with all-*trans*retinoic acid or arsenic trioxide, which transformed the prognosis of this disease. In the non-promyelocytic leukemia, the discovery of key mutations this last decade led to intense efforts to improve therapeutic strategies [42–44]. In parallel, in AML characterized by mutation of the *IDH2* gene, preliminary results of a phase I trial of the IDH2 inhibitor AG-221 showed encouraging rates of response, and nice differentiation of treated leukemic cells *in vitro* and clinically with cases of differentiation syndrome [45]. FLT3 inhibition is one of the major promising targeted therapies in this subset. Previous studies performed with transgenic mice models expressing FLT3-ITD suggested that the expression of this receptor induced myeloproliferative neoplasms rather than AML, suggesting that FLT3-ITD expression by itself did not significantly affect the hematopoietic differentiation process *in vivo* [46]. Our data, as well as those describing the implication of the ERK and of CDK1 in this differentiation, suggest that implication of FLT3-ITD signaling in myeloid differentiation has been underestimated. To date, one of the most potent and clinically advanced FLT3 inhibitor is AC220 (quizartinib) (7, 10–12), and relief of differentiation block *in vitro* and clinically [13] is one of the interesting mechanism of action described for this drug. This pro-differentiation effect of FLT3 inhibition seems to be an on-target effect, as the *in vitro* results were obtained with other FLT3 inhibitors (sorafenib, lestaurtinib and tandutinib) [13–15]. The fact that cases of resistance to quizartinib and other FLT3 inhibitors are unavoidably described further underlines the need to identify downstream targets. To this respect, understanding of signaling and cell cycle molecules impacting hematopoietic differentiation downstream of FLT3-ITD will probably give some keys for future treatments of this AML subtype.

## MATERIALS AND METHODS

### Cell lines and reagents

Human acute myeloid leukemia cell lines MOLM-14 (kindly provided by Martin Carroll, University of Pennsylvania, Philadelphia, PA, USA) MV4-11, K562 (ACC-102 and ACC-10, DSMZ, Braunschweig, Germany) and FLT3-ITD–expressing murine BaF3 cells were cultured in RPMI 1640 medium (Gibco, Life Technologies, Carlsbad, CA, USA) supplemented with 10% fetal bovine serum (Sigma, Saint Louis, CA, USA). TF-1 (ATCC–CRL2003) cells were cultured in RPMI 1640 medium with 10% fetal bovine serum and GM-CSF 2 ng/ml. KG1 and HL-60 (ACC-14 and ACC-3, DSMZ) cell lines were grown in Iscove's Modified Dulbecco's Medium (IMDM, Gibco) with 20% FBS. All cells were grown in the presence of 100 units/ml of penicillin and streptomycin (Invitrogen) at 37°C and 5% CO_2_.

Concerning MOLM-14 cell line, the presence of a monoallelic 21 bp *FLT3*-ITD mutation and of an *MLL*-containing ins(11;9)(q23;p22p23) were verified (Hematology laboratory of Toulouse University Hospital, Prof E. Delabesse and Dr I. Luquet).

The FLT3 inhibitor III [47], Akt inhibitor VIII and STAT5 inhibitor were purchased from Calbiochem (San Diego, CA, USA). The FLT3 inhibitor quizartinib AC220 and the MEK inhibitor PD0325901 were purchased from Selleck Chemicals (Houston, TX, USA). The CDC25 inhibitor IRC-084864 [23] was kindly provided by IPSEN INNOVATION (Marie-Odile Galcera, Les Ulis, France) and NSC-95397 was purchased from Enzo Life Sciences (Farmingdale, NY, USA) [48]. The translation inhibitor cycloheximide was purchased from Sigma (Saint Louis, MO, USA). The proteasome inhibitor bortezomib was kindly provided by Clément Larrue (CRCT Team 18, Toulouse, France). hFLT3 ligand, hGM-CSF, hG-CSF and hIL-3 were purchased from R&D Systems Inc (Minneapolis, MN, USA).

### Patient samples

Patient AML samples used for experiments in Figures 4 and 6 were obtained after informed consent in accordance with the Declaration of Helsinki and stored at the HIMIP collection. According to the French law, HIMIP collection has been declared to the Ministry of Higher Education and Research (DC 2008-307 collection 1) and obtained a transfer agreement (AC 2008-129) after approbation by ethical committees (Comité de Protection des Personnes Sud-Ouest et Outremer II and APHP ethical committee). Clinical and biological annotations of the samples have been declared to the CNIL (Comité National Informatique et Libertés *i.e*. Data processing and Liberties National Committee). Frozen cells were thawed in IMDM medium with 20% FBS and immediately processed for treatment. All patients were diagnosed at the Department of Hematology of Toulouse University Hospital. Their characteristics are summarized in Table 1.

### Co-cultures

Patient samples, containing at least 80% of blasts, were co-cultured with human stromal cells (HS-5 (ACC-441) kindly provided by Helena Boutzen, CRCT Team 18, Toulouse, France) in IMDM (Gibco) supplemented with 15% BIT (Stem Cell Technologies, Vancouver, BC, Canada), 100 units/ml penicillin and streptomycin (Invitrogen), 5 μM β-mercaptoethanol (Invitrogen), 1 mM pyruvate (Sigma), MEM 1x (Sigma), 100 ng/mL DNase (MP Biomedicals, Solon, OH, USA), 10 ng/mL hIL-3, 100 ng/mL hSCF, and 10 ng/ml hTPO (all from R&D Systems Inc, Minneapolis, MN, USA). All the samples were then processed for treatment with the different reagents (IRC-083864 or NSC-95397).

### siRNA

The MV4-11 cell line was transfected with the Amaxa nucleofection technology (Lonza, Koeln, Germany). Cells (2 × 10^6^) were resuspended in 100 μL of Amaxa solution L. 300 nM of specific STAT5A and STAT5B siRNA (ON-TARGETplus SMARTpool, human STAT5A and STAT5B, Dharmacon) or total CDC25A siRNA (Hs_CDC25A_9, Qiagen, Hilden, Germany) or 3′UTR CDC25A siRNA (CDC25A 2943, Sigma) or negative control (si genome control pool non targeting #2, or ON-TARGETplus control pool (Dharmacon)) were added, and cells were transfected with the nucleofector device (program Q-001; solution V and program O-017 for MOLM-14; solution R and program V-001 for KG1). Cells were subsequently resuspended in normal culture medium at a concentration of 5 × 10^5^ cells/mL. Twenty-four or forty-eight hours after transfection, cells were counted (trypan blue staining), and western blotting was performed.

### Lentiviral infections

Lentiviral shRNA particules were generated as previously described [49]. Briefly, we used HEK-293-T packaging cells, co-transfected with lentiviral protein (GAG, POL, and ENV) encoding plasmids, and MISSION^®^ shRNA lentiviral plasmids (pLKO.1-puro: non-targeting shRNA control vector and shCDC25A vector: TRCN10704NM_001789.x-529s1c1TRC1 (Sigma), separately). Supernatants containing lentivirus were collected 48 h after transfection, during 3 consecutive days. MOLM-14 cells were plated at 5 × 10^5^ cells/mL in serum-free medium and 20 μl of lentiviral supernatant was added during 6 h. Cells were then grown in 10% FBS RPMI medium.

### Western blot

2 × 10^6^ cells were usually lysed in 100 μL of NuPAGE^®^ LDS Sample Buffer (Novex, Life Technologies, Carlsbad, CA, USA), sonicated for 15 seconds, and boiled for 3 minutes. Proteins were then resolved on NuPAGE^®^ 4–12% Bis-Tris Gels and transferred to nitrocellulose membrane. Saturation of the membrane was done for 1 hour in Tris Buffer Saline with Tween 0.1% (TBS-T) containing 5% non-fat milk or 5% bovine serum albumin. Membranes were blotted with proper antibodies overnight at 4°C, washed thrice with TBS-T, and incubated for 30 minutes with HRP-conjugated secondary antibody (Promega, Madison, WI, USA). After three additional washes, detection was achieved with Supersignal West Pico Chemiluminescent substrate (Thermo Fisher Scientific, Rockford, IL, USA). The antibodies used were: monoclonal anti-CDC25A (F-6), anti-Cyclin D1 (HD11), anti-Pim1 (12H8), anti-c-myc (9E10), and polyclonal anti-CDC25B (C-20), anti-CDC25C (C-20), anti-Cyclin A (C-19), and anti-Akt1/2/3 (H-136), from Santa Cruz Biotechnology (Santa Cruz, CA, USA), monoclonal anti-phospho-p44/42 MAPK (Erk1/2) (Thr202/Tyr204) (E10) and polyclonal anti-phospho-STAT5 (Tyr 694), anti-STAT5, anti-p44/42 MAPK, anti-phospho-Ser473 Akt (D9E) XP, anti-phospho-C/EBPα (Ser21), anti-C/EBPα (p42) and anti-C/EBPε (C-22), from Cell Signaling Technology (Beverly, MA, USA), anti-p27^KIP1^ from BD Biosciences (San Diego, CA, USA); anti-β-actin and anti-α-tubulin from Sigma.

### Quantitative RT-PCR

Total RNA was extracted by RNeasy Kit (Qiagen) according to the manufacturer. RNA quality and purity was assessed by using the Agilent RNA 6000 Nano kit (Agilent Technologies, Santa Clara, CA, USA). cDNA was generated with the SuperScript III First-Strand Synthesis System for RT-PCR (Invitrogen) following the manufacturer instructions. The PCR was performed with TaqMan^®^ Gene Expression Master Mix (Applied Biosystems, Foster City, CA, USA) with 1 μl of cDNA on a LightCycler^®^480 (Roche). The primer used was Hs00947994_m1 (Applied Biosystem) for *CDC25A*. *GUSB* (Hs00939627_m1) and *B2M* (Hs00984230_m1) were used as housekeeping genes. Results were analyzed with the LightCycler^®^480 software release 1.5.0 SP4 using the conventional ΔΔCt method.

### Flow cytometry

Apoptotic cells were detected with Annexin V-FITC detection kit from BD Pharmingen (San Diego, CA, USA) according to the manufacturer instructions. To evaluate AML cell differentiation, cells were stained for 30 min with the following anti-human monoclonal antibodies: CD11b-PE (Beckman Coulter), CD14-FITC (BD Biosciences), CD15-APC (BD Biosciences). For patient samples, additional hCD45-APC-H7 (BD Biosciences), Annexin V-Pacific Blue (BioLegend, San Diego, CA, USA) and 7-AAD (Sigma) were used. Data were collected on a LSRII or a LSRFortessa cytometer (BD Biosciences), and analyzed with FlowJo software. A minimum of 10,000 events was collected.

### Clonogenic assay

AML cells (10^6^/mL) were grown in duplicate in H4230 methylcellulose medium (Stem Cell Technologies) supplemented with 10% 5637-conditionned medium as described [50]. IRC-083864 was added at increasing concentrations in the culture medium. Cells were incubated for 7 days in a humidified CO_2_ incubator. Leukemic colonies were then scored under an inverted microscope.

### Morphological examination

10^5^ cells were spun at 500 rpm for 5 minutes onto glass slides and May-Grünwald-Giemsa stained at the Toulouse University Hospital hematology laboratory.

### Statistics

Experiments in cell lines were performed at least 3 times. Results are expressed as mean value +/− SEM. Statistical analysis of the data was performed by the Mann-Whitney *U* test and two-way Anova for multiple comparisons using GraphPad Prism software, version 5.0 (GraphPad Software Inc., La Jolla, CA). Differences were considered as significant for *p* values < 0.05 (*), *p* < 0.01 (**), *p* < 0.001 (***).

## SUPPLEMENTARY METHODS FIGURES AND TABLES


