# Supplementary material for: CDC25A governs proliferation and differentiation of FLT3-ITD acute myeloid leukemia
Source: Oncotarget. 2015 Oct 16;6(35):38061–78. doi: 10.18632/oncotarget.5706 (PMC4741984; doi:10.18632/oncotarget.5706)
Supplement: Supplementary file 1 [file oncotarget-06-38061-s001.pdf]

SUPPLEMENTARY METHODS

Patients

Between Jan 1st, 2000, and Dec 31st, 2010, 513 consecutive patients (65 years of age or younger) with a new diagnosis of AML have been treated by intensive chemotherapy in Toulouse University Hospital. Diagnosis workup, treatment modalities and outcome have been described elsewhere (Bertoli S *et al*. Blood 2013). Among this cohort of patients, 188 samples stored after informed consent in the HIMIP tumor bank of the U1037 Inserm department (n°DC-2008-307-CPTP1 HIMIP) were available for this study. *CDC25A* mRNA expression was assessed in each sample using Fluidigm technology. 151 had data on clonogenic properties at diagnosis (clonogenic assay described in the Methods section). We restricted our analysis to the intermediate cytogenetic group according to the Medical Research Council classification (Grimwade D *et al*. Blood 2010) (n = 100); the initial characteristics are depicted in Supplementary Table S1. *FLT3*-ITD mutational status was known for 94 of them: 35 were mutated and 59 were wild-type. Their characteristics are depicted in Supplementary Tables S2 and S3 respectively. This study was approved by the institutional review board (Ethical Committee of Research). According to the French law, HIMIP collection has been declared to the Ministry of Higher Education and Research (DC 2008-307 collection 1) and obtained a transfer agreement (AC 2008-129) after approbation by the "Comité de Protection des Personnes Sud-Ouest et Outremer II" (ethical committee). Clinical and biological annotations of the samples have been declared to the CNIL (Comité National Informatique et Libertés *i.e*. Data processing and Liberties National Committee).

Total RNA extraction and reverse transcription-PCR

Total RNA were extracted from frozen cells (7 to 15 million of cells) stored in 1 ml of Tri Reagent RNA/DNA/protein isolation reagent (Molecular Research Center). RNA concentration has been determined using the NanoDrop ND-1000 spectrophotometer (NanoDrop Technologies Inc, Thermo Scientific, Brebières, France). RNA quality and purity was assessed on the Agilent 2100 BioAnalyser by using the Agilent RNA 6000 Nano kit (Agilent Technologies, Santa Clara, CA, USA). Only RNA presenting a RIN>6.5 were selected for expression analysis (Around 90% of the samples have a RIN>8). cDNA was generated from 1 µg of RNA with the SuperScript VILO cDNA Synthesis Kit (Invitrogen) for reverse transcription-PCR following the manufacturer's suggestions. In order to ensure good quality reverse transcription step, one part of each cDNA was used to check ABL1 ("TaqMan Gene Expression Assay", Applied Biosystems, Hs01104728_m1) expression level homogeneity using ABI Prism 7300 HT (Applied Biosystems, Carlsbad, CA, USA).

Specific target amplification and quantitative PCR

The other part of each cDNA was diluted in water to 5 ng/µl and dedicated to target amplification for Biomark Dynamic Arrays (Fluidigm, BioMark, Pueblo, CO, USA). Inventoried TaqMan assays (Applied Biosystems) were pooled using 84 probes and primers pairs, to a final concentration of 0.2X for each of the 84 assays. To increase sensitivity, a multiplexed preamplification process was performed for the pool on every 1.25 µl cDNA using 14 cycles cDNA preamplification step (95°C for 15s and 60°C 4 min) and Taqman PreAmp Master Mix (Applied Biosystems) in a standard PCR Thermocycler. Preamplified cDNA was diluted 1:5 in 10 mmol/L Tris, 1 mmol/L EDTA. Diluted cDNA (2.25 µl) was added to 2.5 µl Taqman Universal PCR Master Mix (Applied Biosystems) and 0.25 µl GE Sample Loading Reagent (Fluidigm). In a separate tube, 3.5 µl of Taqman Assay was added to 3.5 µl Sample Loading Reagent. 5 µl cDNA samples were loaded into the sample inlet wells, and 5 µl assay samples were loaded into assay detector inlets. Because 188 samples were to be analyzed in duplicated, five 96.96 Dynamic Arrays (Fluidigm) were used. For each plate, 1 well was loaded with H_20 as control for contamination. Genomic DNA (gDNA) from 3 different patients was loaded in order to check if TaqMan assays can also amplify genomic DNA. To verify STA efficiency, a sample control gDNA and assay control RNAse P Taqman probe was treated (Lifetech PN 4316844), preamplified and quantified using the same mastermix. The expected value cq was between 12 and 13. In order to do inter-plate calibration, a sample calibrator made of cDNA from the patient #1 was included in duplicate in each plate. The chip was primed and placed into the NanoFlex Integrated fluidic circuit controller where 8 nl of cDNA and 1 nl of Assay were mixed. Real time PCR analysis was completed on the BioMark System (Fluidigm).

Data processing

Raw data obtained from the system's software using the auto detector function in order to establish the threshold setting (BioMark Realtime PCR Analysis V2.1.1, Fluidigm), were checked using the graphical representation of the plate layout. Among all reactions investigated, 0 were rejected due to bubbles or instable

ROX signal. All amplification curves were displayed for each well of the calibrator sample. When threshold for cycle did not meet quality criteria (*i.e.* threshold set in the linear phase of the amplification curve instead of the exponential phase), threshold value was set manually. The threshold established for the first Dynamic Array were applied to the 4 other Dynamic Arrays. Wells with very high (>26), absent (999) or very low (<2) endogenous Ct resulted in exclusion.

Normalization method

In order to perform the real time qPCR data normalization with the housekeeping genes and the inter-plate calibration have been performed by using the qbase+ algorithm as described (Hellemans J *et al*., RNA, 2007). Among the 9 housekeeping genes tested (GUSB (Hs99999908_m1), ACTB (Hs99999903_m1), ABL1 (Hs01104728_m1), G6PD (Hs00166169_m1), TBP (Hs00427621_m1), GAPDH (Hs03929097_g1), HMBS (Hs00609293_g1), B2M (Hs00984230_m1), UBC (Hs00824723_m1)), GeNorm algorithm determined the four most stable which were GAPDH, GUSB, TBP and ABL1 and calculate the gene expression normalization factor. Expression values are given in ΔΔCt.

Applied biosystems reference

CDC25A: Hs00947994_m1

Statistical analysis

We categorized gene expression in a binary variable “high expression” *versus* “low expression” from the median expression value. Patients’ characteristics have been compared according to the gene expression level using Mann-Whitney *U* test for quantitative data and Fisher’s exact test for qualitative data.

FLT3-ITD/TKD cell line

The *FLT3-ITD* gene was cloned into the pLKO.1-blast lentiviral expression vector (Addgene Plasmid 26655) (Stewart SA *et al*, 2003). Mutation producing a D835Y amino-acid substitution within FLT3 kinase domain (FLT3-ITD-D835Y) was generated using the QuikChange II XL Site-Directed Mutagenesis Kit (Agilent Technologies, Santa Clara, CA, USA), in accordance with the manufacturer’s instructions using the following (5′-3′) primer: CTTTGGATTGGCTCGATATATCATGAGTGATTCCA AC.

We used 293-T packaging cells to produce FLT3-ITD and FLT3-ITD-D835Y lentivirus through co-transfection of FLT3-containing plasmids with lentiviral protein-encoding plasmids. Supernatants were collected over three consecutive days beginning 48 h post-transfection, and stored at –80°C.

We plated 10^6/ml MOLM-14 cells in 100 µl of alpha-MEM medium and added 5 µl of lentiviral supernatant to the culture. After 3 h, culture medium was supplemented with 10% FBS. Puromycin selection started 48 h after lentiviral infection and allowed enrichment for FLT3-ITD or FLT3-ITD-D835Y-expressing MOLM-14 cells.

REFERENCES

Bertoli S, Berard E, Huguet F, Huynh A, Tavitian S, Vergez F, Dobbelstein S, Dastugue N, Mansat-De Mas V, Delabesse E, Duchayne E, Demur C, Sarry A, et al. Time from diagnosis to intensive chemotherapy initiation does not adversely impact the outcome of patients with acute myeloid leukemia. Blood. 2013; 121:2618–2626.

Grimwade D, Hills RK, Moorman AV, Walker H, Chatters S, Goldstone AH, Wheatley K, Harrison CJ, Burnett AK. Refinement of cytogenetic classification in acute myeloid leukemia: determination of prognostic significance of rare recurring chromosomal abnormalities among 5876 younger adult patients treated in the United Kingdom Medical Research Council trials. Blood. 2010; 116:354–365.

Hellemans J, Mortier G, De Paepe A, Speleman F, Vandesompele J. qBase relative quantification framework and software for management and automated analysis of real-time quantitative PCR data. Genome biology. 2007; 8:R19.

Stewart SA, Dykxhoorn DM, Palliser D, Mizuno H, Yu EY, An DS, Sabatini DM, Chen IS, Hahn WC, Sharp PA, Weinberg RA. Novina CD Lentivirus-delivered stable gene silencing by RNAi in primary cells. RNA. 2003; 9:493–501.

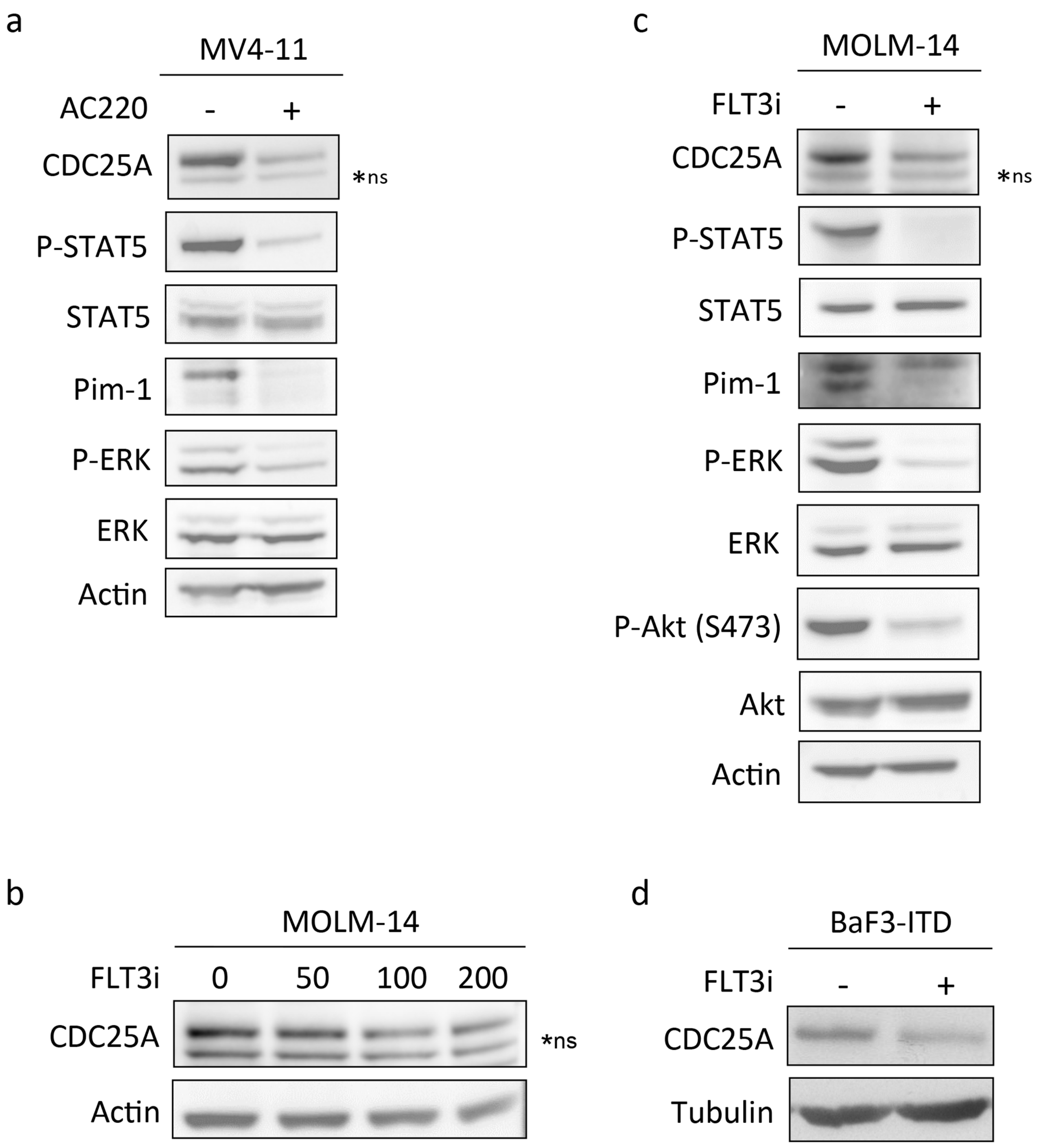

Supplementary Figure S1: CDC25A is an early cell cycle target downstream of FLT3-ITD. a. MV4-11 cells were treated for 4 hours with the second generation FLT3 inhibitor AC220 (quizartinib, 2 nM) and the expression level of CDC25A, as well as STAT5, ERK and Pim1 protein expression and phosphorylation levels were analyzed. **b.** MOLM-14 cells were treated with increasing concentrations of FLT3 inhibitor III, and CDC25A protein level was followed by western blot. **c.** MOLM-14 cells were treated with FLT3 inhibitor III for 2 hours, and CDC25A protein as well as ERK, STAT5 and Akt activation levels were analyzed. These results are representative of three independent experiments. Actin was used as a loading control. ns: non specific. **d.** BaF3 murine cell line stably expressing FLT3-ITD (BaF3-ITD) was treated for 5 hours with FLT3 inhibitor III (100 nM), and the expression level of CDC25A was analyzed by western blot. Tubulin was used as a loading control.

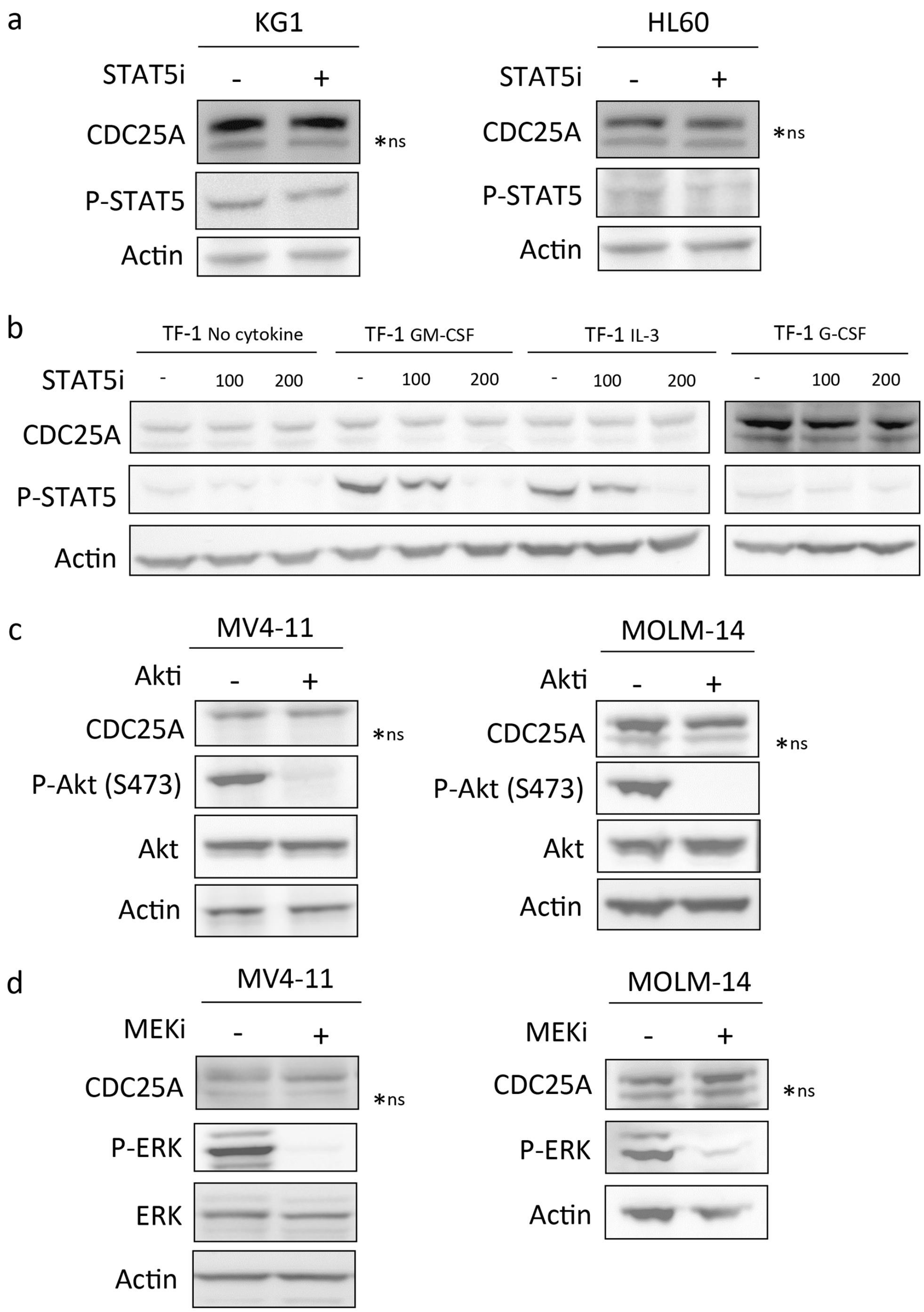

Supplementary Figure S2: STAT5 inhibition does not induce CDC25A down-regulation in FLT3 WT cells and Akt or ERK inhibition does not induce CDC25A down-regulation downstream of FLT3-ITD. a. KG1 (left panel) and HL60 cells (right panel) were treated for 2 hours with STAT5 inhibitor (100 nM). CDC25A and STAT5 phosphorylation levels were analyzed by western blot. **b.** TF-1 cells were washed from their GM-CSF containing medium and then cultured in RPMI medium containing either GM-CSF 10 ng/mL, IL-3 10 ng/mL, G-CSF 10 ng/mL or no cytokine. They were then treated for 3 hours with STAT5 inhibitor (100 nM or 200 nM). CDC25A and STAT5 phosphorylation levels were analyzed by western blot. (c-d) MV4-11 and MOLM-14 cells were treated with **c.** Akt inhibitor VIII (5 µM) or **d.** MEK inhibitor PD0325901 (50 nM) for 2 hours, and the effect on CDC25A protein level was analyzed by western blot. The efficiency of these inhibitors was verified by western blot analysis of Akt and ERK phosphorylation levels. These results are representative of three independent experiments. Actin was used as a loading control. ns: non specific.

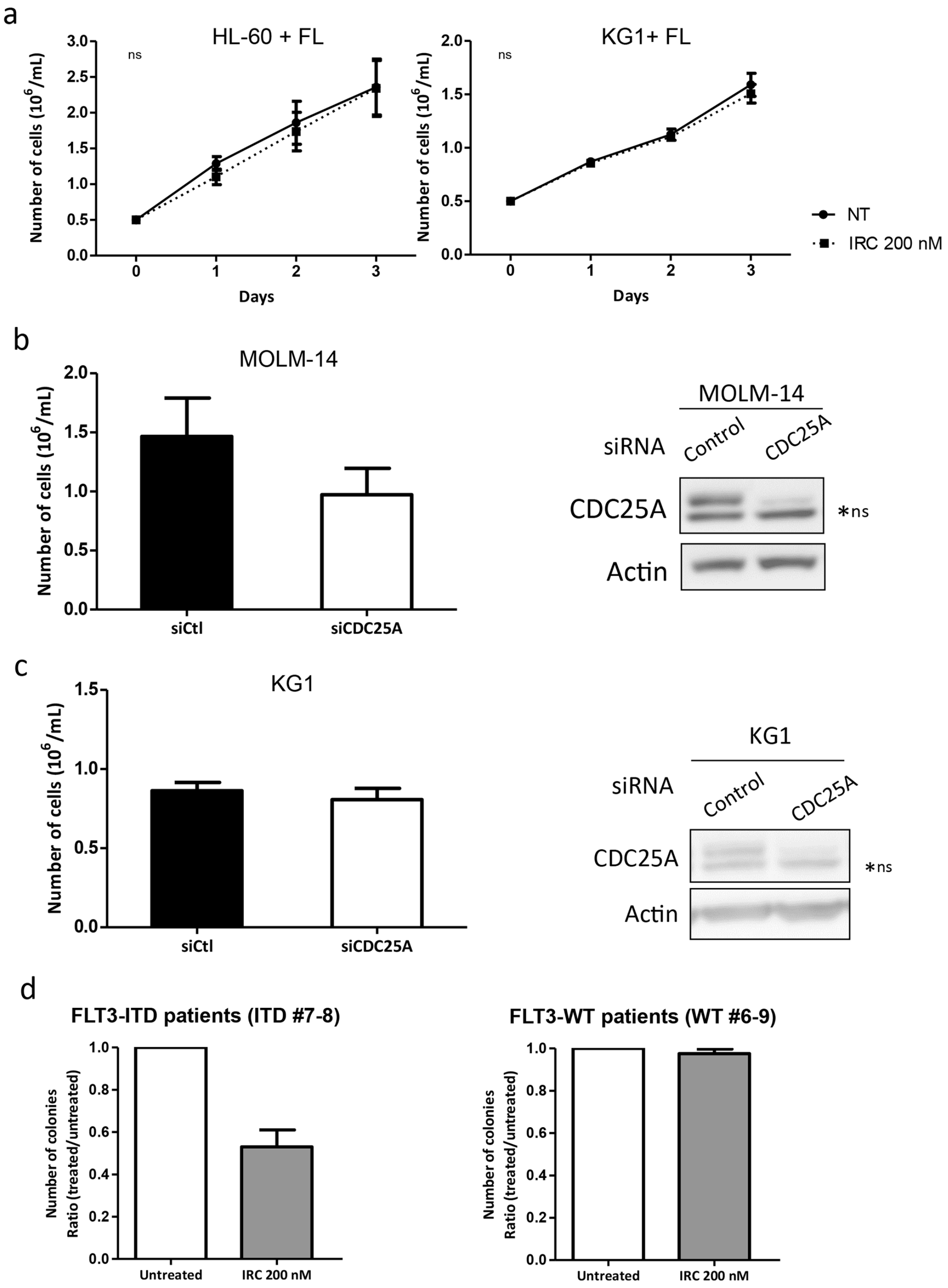

Supplementary Figure S3: CDC25A is an important determinant of FLT3-ITD leukemic cells proliferation. a. KG1 and HL-60 FLT3 wild type cells were cultured in the presence of the CDC25 inhibitor IRC-083864 (200 nM) upon stimulation by FLT3 ligand (5 ng/mL). Cells were harvested each day and counted after trypan blue staining. The graph represents three independent experiments. **b.** MOLM-14 cells were transfected for 24 hours with an siRNA against the 3′-UTR sequence of CDC25A, different from the siRNA used in Figure 4c. Cells were counted (left panel) and CDC25A protein level was followed by western blot (right panel), as described in Figure 4c. **c.** KG1 cells were transfected with CDC25A siRNA for 48 hours, in the same conditions than MV4-11 and MOLM-14 cells in Figure 4c. Cells were then counted (left panel), and CDC25A level was estimated by western blot (right panel). **d.** Primary cells from patients were cultured in semi-solid medium to estimate their clonogenic potential in a medium with recombinant human growth factors : methylcellulose with IMDM 20% FCS, GM-CSF: 10 ng/mL, FLT3 ligand: 50 ng/mL and IL-3: 10 ng/mL, and treated with IRC-083864 (200 nM). 2 FLT3-ITD positive (upper panel) and 4 FLT3-wild type (lower panel) AML primary samples were used for these experiments. Leukemic colonies were scored under an inverted microscope at day 7.

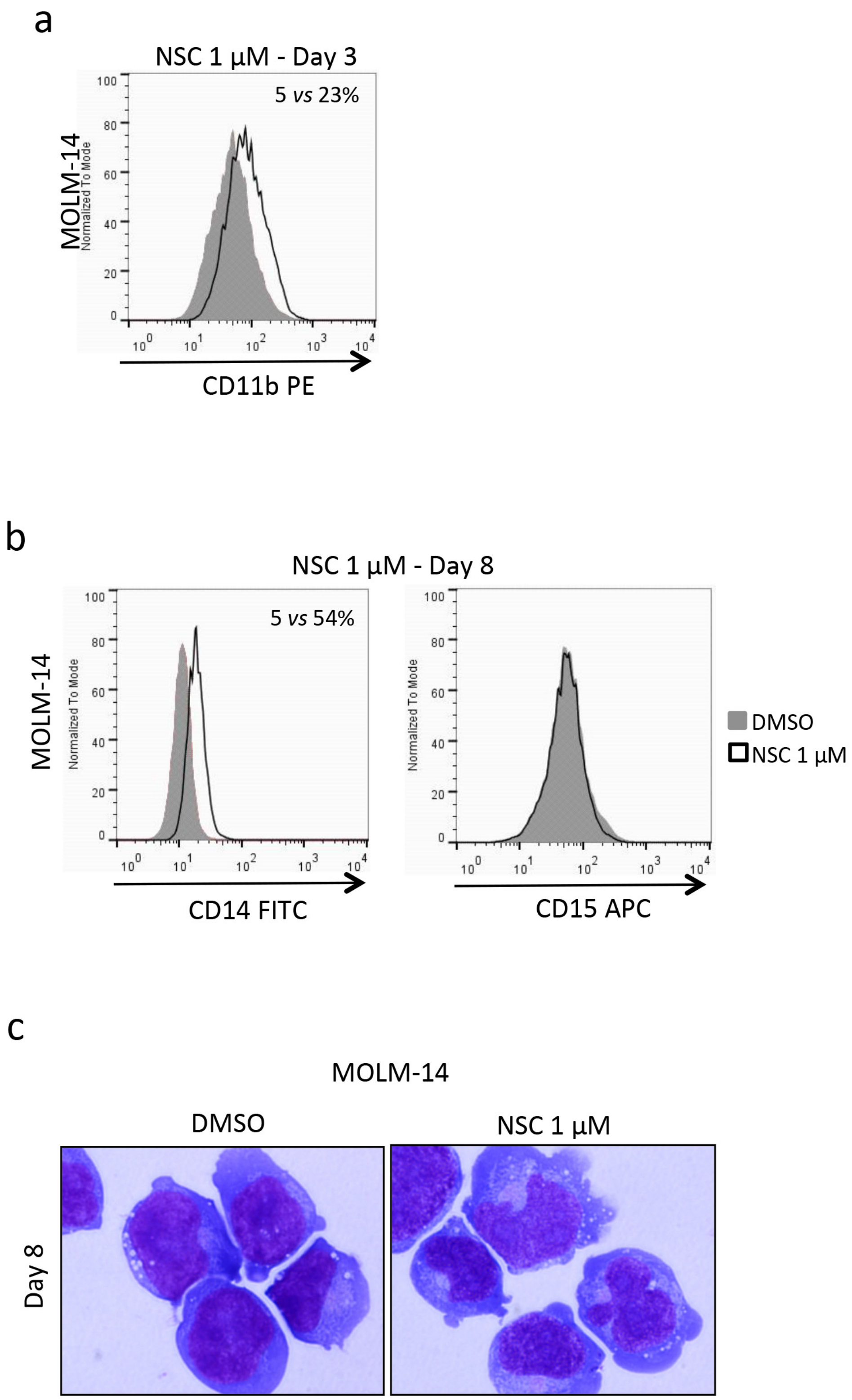

Supplementary Figure S4: CDC25 inhibition with NSC-95397 relieves differentiation block in MOLM-14 cells. MOLM-14 cells were treated for different times with NSC-95397 (1 μM) and cells were analyzed for the expression of CD11b, CD14 and CD15 by flow cytometry a–b. and for morphological modifications by microscopy **c.** Original magnification x100. These results are representative of three independent experiments.

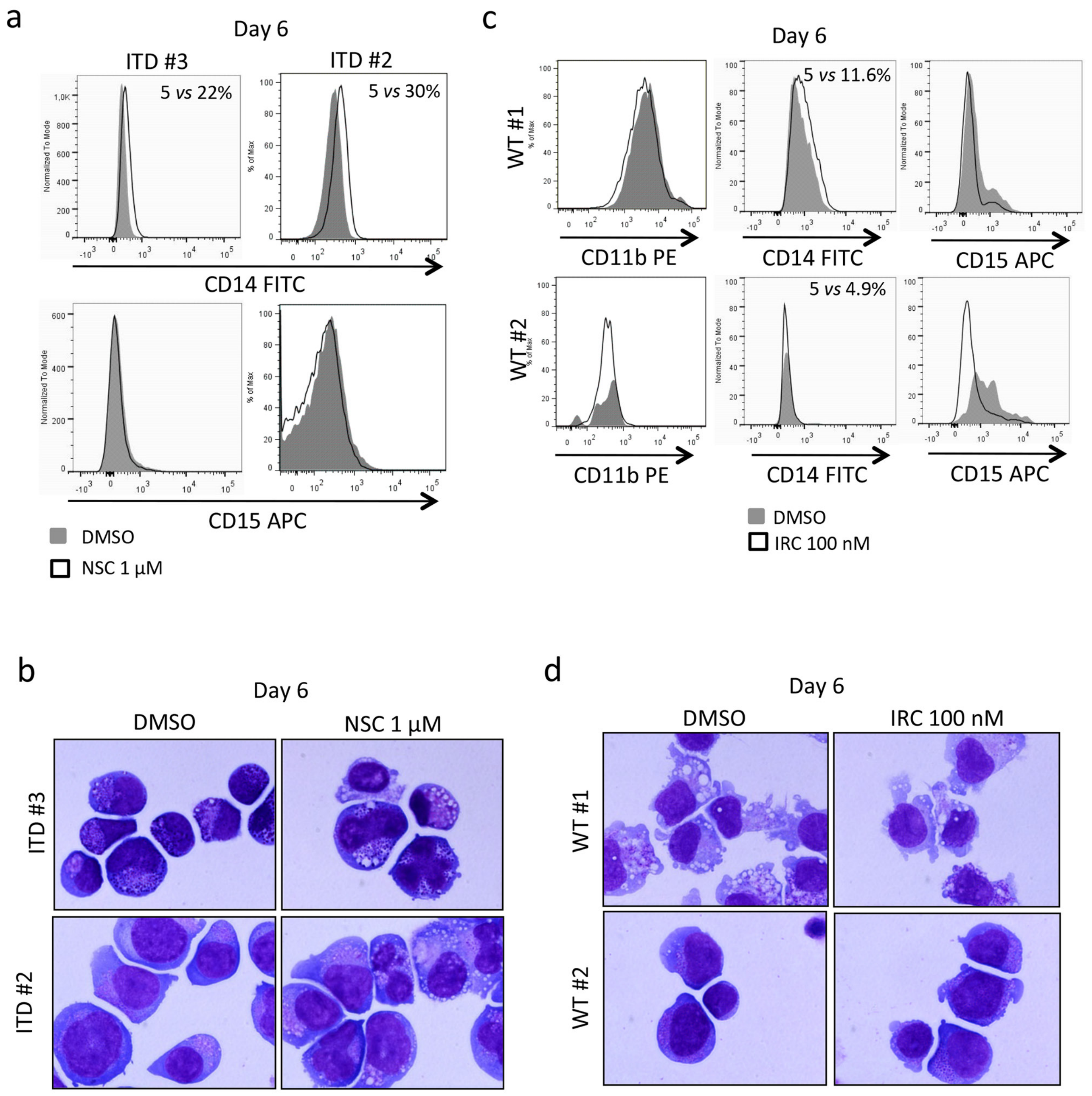

Supplementary Figure S5: CDC25 inhibition relieves differentiation block in FLT3-ITD but not in FLT3wt AML primary cells. a–b. CD14 and CD15 flow cytometry expression analysis (a) and morphological analyses (b) of two primary samples (ITD #2–3) expressing FLT3-ITD treated with the CDC25 inhibitor NSC-95397 (1 µM) for 6 days. Original magnification x100. **c–d.** CD11b, CD14 and CD15 flow cytometry expression analysis (c) and morphological analyses (d) of two primary samples (WT #1–2) expressing wild type FLT3 treated with the CDC25 inhibitor IRC-083864 (100 nM) for 6 days. Original magnification x100.

Supplementary Table S1: Patients characteristics

		Total cohort n = 100	Low CDC25A n = 59	High CDC25A n = 41	p
**Age – years**	**Median (IQR)**	49 (40–57)	52 (44–58)	42 (35–55)	0.02$^{\%}$
**AML status – n (%)**	***De novo***	90 (90)	54 (92)	36 (88)	ns$^{\#}$
	**Secondary**	10 (10)	5 (8)	5 (12)	
**White blood cell count (10^9/L)**	**Median (IQR)**	36 (12–86)	51 (17–100)	18 (5–46)	0.007$^{\%}$
**Mutations – n (%)**	***NPM1c***	45/93 (48)	36/56 (64)	9/37 (24)	<0.001$^{\#}$
	***FLT3*-ITD**	35/94 (37)	22/56 (39)	13/38 (34)	ns$^{\#}$
	CEBPA	17/83 (21)	8/52 (15)	9/31 (29)	ns$^{\#}$

IQR: interquartile range.
Comparisons between low CDC25A and high CDC25A subgroups were performed using $^{\%}$ Mann-Whitney *U* test and $^{\#}$ Fisher's exact test.

Supplementary Table S2: Patients characteristics in *FLT3*-ITD mutated subgroup

		Total n = 35	Low CDC25A n = 22	High CDC25A n = 13	p
**Age – years**	**Median (IQR)**	46 (41–55)	51 (43–56)	41 (36–49)	ns$^{\%}$
**AML status – n (%)**	***De novo***	35 (100)	22 (100)	13 (100)	ns$^{\#}$
	**Secondary**	0	0	0	
**White blood cell count (10^9/L)**	**Median (IQR)**	52 (17–111)	69 (37–112)	35 (12–87)	ns$^{\%}$
**Mutations – n (%)**	***NPM1c***	24/35 (69)	19/22 (86)	5/13 (39)	<0.01$^{\#}$
	CEBPA	2/28 (7)	0/18	2/10 (20)	ns$^{\#}$
**ITD allelic ratio**	**Median (IQR)**	0.40 (0.2–0.8)	0.41 (0.2–0.8)	0.27 (0.1–0.7)	ns$^{\%}$
**ITD length (bp)**	**Median (IQR)**	52.5 (30–62)	51 (29–60)	52.5 (30–63)	ns$^{\%}$

IQR: interquartile range; bp: base pair.
Comparisons between low CDC25A and high CDC25A subgroups were performed using $^{\%}$ Mann-Whitney *U* test and § Fisher's exact test.

Supplementary Table S3: Patients characteristics in *FLT3*-wild type subgroup

		Total n = 59	Low *CDC25A* n = 34	High *CDC25A* n = 25	p
**Age – years**	**Median (IQR)**	49 (39–57)	52 (46–59)	42 (33–53)	0.02[%]
**AML status – *n* (%)**	***De novo***	50 (85)	30 (88)	20 (80)	ns[#]
	**Secondary**	9 (15)	4 (12)	5 (20)	
**White blood cell count (10^9/L)**	**Median (IQR)**	31 (8–69)	42 (17–82)	19 (4–45)	0.03[%]
**Mutations – *n* (%)**	***NPM1c***	21/59 (36)	16/33 (49)	5/26 (19)	0.03[#]
	CEBPA	15/54 (28)	8/34 (24)	7/20 (35)	ns[#]

IQR: interquartile range.
Comparisons between low *CDC25A* and high *CDC25A* subgroups were performed using [%] Mann-Whitney *U* test and [§]Fisher's exact test.
